# Identifying Facial Features and Predicting Patients of Acromegaly Using Three-Dimensional Imaging Techniques and Machine Learning

**DOI:** 10.3389/fendo.2020.00492

**Published:** 2020-07-29

**Authors:** Tian Meng, Xiaopeng Guo, Wei Lian, Kan Deng, Lu Gao, Zihao Wang, Jiuzuo Huang, Xiaojun Wang, Xiao Long, Bing Xing

**Affiliations:** ^1^Department of Plastic and Aesthetic Surgery, Peking Union Medical College Hospital, Chinese Academy of Medical Sciences & Peking Union Medical College, Beijing, China; ^2^Department of Neurosurgery, Peking Union Medical College Hospital, Chinese Academy of Medical Sciences & Peking Union Medical College, Beijing, China; ^3^China Pituitary Disease Registry Center, Beijing, China; ^4^China Pituitary Adenoma Specialist Council, Beijing, China

**Keywords:** acromegaly, three-dimensional imaging, machine learning, facial changes, early detection, prediction

## Abstract

**Objective:** Facial changes are common among nearly all acromegalic patients. As they develop slowly, patients often fail to notice such changes before they become obvious. Consequently, diagnosis and treatment are often delayed. So far, convenient and accurate early detection of this disease is still unavailable. This study is designed to combine the use of 3D imaging and machine learning techniques in facial feature analysis and identification of acromegalic patients, in an effort to ascertain how both techniques performed in terms of applicability and value in the early detection of the disease.

**Methods:** One hundred and twenty-four participants including 62 patients with acromegaly and 62 matched controls were enrolled. Using three-dimensional imaging techniques, 58 facial parameters were measured on each face. A two-way analysis of variance (ANOVA) and a *post-hoc t*-tests were conducted to examine the variations of these parameters with disease status and gender. Using linear discriminant analysis (LDA), we further distinguished patients from controls, characterized what combinations of the parameters could best predict disease state and their relative contributions.

**Results:** Patients are significantly different from normal subjects in many variables, and facial changes of male patients are more significant than female ones. Both male and female patients present following major changes: the increase of facial length and breadth, the widening and elevation of the nose, the thickening of vermilion and the enlargement of the mandible. Facial variables which strongly related to the pathological states can be used to predict the morbid state with high accuracy (prediction accuracies 92.86% in females, *p* < 0.0001 and 75% in males, *p* < 0.001). We have further testified that only a few variables play a vital role in disease prediction and the vital combination of variables vary with gender.

**Conclusions:** Three-dimensional imaging enables comprehensive and accurate quantification of facial characteristics, which makes it a promising technique to investigate facial features of acromegalic patients. In combination with machine learning technique, patients can be accurately identified and predicted by their facial variables. This approach might be beneficial for the early detection of acromegalic patients and timely consultation to improve their outcomes.

## Introduction

Acromegaly is a relatively rare chronic disease, and is now thought to affect 28–137 individuals per million people (1, 2). Up to 99% of patients with acromegaly harbor a pituitary somatotroph adenoma, leading to growth hormone (GH) and Insulin-like growth factor 1 (IGF-I) hypersecretion, resulting in multi-system implications (3, 4). The disease is associated with increased morbidity and mortality, especially in undiagnosed, untreated conditions, which lead to prolonged durations (5–7).

The most common complaints and clinical manifestations of this disease are facial changes and acral growth (8) that, however, occur very slowly and are always noticed only after the changes become significant. Therefore, the insidious trait of the disease often causes a diagnostic delay for an average of 4–6 years (6). Admittedly, early detection of the patients' facial changes is very helpful for the early identification of suspected patients as well as the early diagnosis and treatment. However, convenient and accurate early detection methods for the disease remain unavailable.

Facial changes have been used as one of the diagnostic criteria of acromegaly. However, there are no specific quantitative diagnostic criteria (9–11). At present, recognition of facial features of acromegalic patients relies, to a large extent, on the clinical experience of endocrinology specialists. The disease, however, is often neglected by patients and inexperienced physicians. Since there has been a lack of research data for the disease, disease-related facial information of the patients often fails to be utilized effectively. By reviewing the current studies on facial morphological changes in acromegalic patients, it was found that cephalometry has been used in most studies. Because of methodological limitations, many facial parameters cannot be measured and analyzed. Therefore, it is impossible to comprehensively summarize the facial changes of acromegalic patients. In recent years, some studies have automatically distinguished patients from non-patients using 2D photographs combined with computer software. Although no specific parameters have been analyzed, these studies show that facial changes in acromegalic patients are characteristic and can be classified and identified by machines or clinicians (12–15).

In the research group's previous study, references were made from the existing literature to preliminarily select 14 basic facial parameters that can be used to characterize the facial contour, nose, and lips, before making precise measurements using 3D imaging, and analyzing their correlations with hormone levels (16). Because of the complexity and diversity of facial information, 3D imaging was further used in this study to comprehensively mine the facial information available in more subjects, and analyze their internal correlations. A lot of information pertaining to this method has not been considered by traditional methods. This study aims to better defining the distinctive facial features associated with the disease, so as to help early detection and identification of patients, and provide references for further formulation of diagnostic criteria for the patients' facial morphological changes.

## Materials and Methods

### Study Design and Participants

This is a 1:1 matched cross-sectional study.

Sixty-two patients (34 males, 28 females), who were newly diagnosed with acromegaly and admitted to the neurosurgical ward of Peking Union Medical College Hospital for surgery, were consecutively included in this study. The self-reported disease durations were 18–144 months, with an average of 78 months. The diagnosis of acromegaly was based on clinical manifestations and biochemical assessments, along with pituitary magnetic resonance imaging (MRI) (9–11). All participants have prominent increase in hormone levels (serum GH range from 2.87 to 174 ng/ml; serum IGF-1 range from 394 to 1447 ng/ml), imageological confirmed pituitary masses, as well as varying degrees of facial characteristic changes.

Sixty-two controls (34 males, 28 females) were recruited from the outpatient clinic of Department of Plastic and aesthetic surgery, Peking Union Medical College Hospital, and were matched by age, gender, height, weight, and physiognomic characters to the patients. None of the healthy controls has maxillofacial or occlusal malformations, or other conditions that might affect the appearance, and were not seeking maxillofacial surgery at the clinic. On the grounds of practical experience and existing knowledge, we included controls whose weight (or body weight index, BMI) can be equal to or a little bit less than, but not greater than their paired patients. Subjects who have extreme or paradoxical facial features compared to patients were excluded.

The study was approved by the Institutional Review Board at Peking Union Medical College Hospital, Chinese Academy of Medical Sciences (Ethical Number: ZS-1324). All research was performed in accordance with relevant guidelines and regulations. Informed consent for both study participation and publication of identifying information/images in an online open-access publication was obtained from each participant. All participants were Chinese Han people.

### Facial Images Acquisition and Processing

#### 3D Images Acquisition

Photos were taken using VECTRA H1 handheld imaging system (Canfield Scientific, Inc. USA). Patients were asked to sit straight and still, with relaxed expression, eyes gaze ahead, and mouth closed. The patient's hair, clothing and jewelry were secured away from the face, ears, and neck. Facial images were captured at three different angles for each subject. First, the photographer positioned the camera directly in front of the subject, aimed the green dots between the upper lip and nose. After converging the green dots to a single point by adjusting camera distance from the subject, the first image was captured. Then, the camera was positioned at a 45°angle from the front to either the lateral side of the face. After aiming and converging the green dots at the middle of the cheek, the second and third images were captured. The three images were then stitched together into a single 3D image using VECTRA software (Canfield Scientific, Inc. USA). Compared with traditional 2D photos, the advantage of 3D images is that it is like a three-dimensional casting that is 1: 1 as the exact same size of the subject and stored in the computer. Researchers can retrieve images at any time, select landmarks on images, and perform various measurements, including linear distances, curve distances, angles, circumferences, area and volume, etc. The measured value is isometrical to the photographed object, no conversion is required, and the accuracy is 0.001 mm.

#### Images Processing

**Figure 1:**
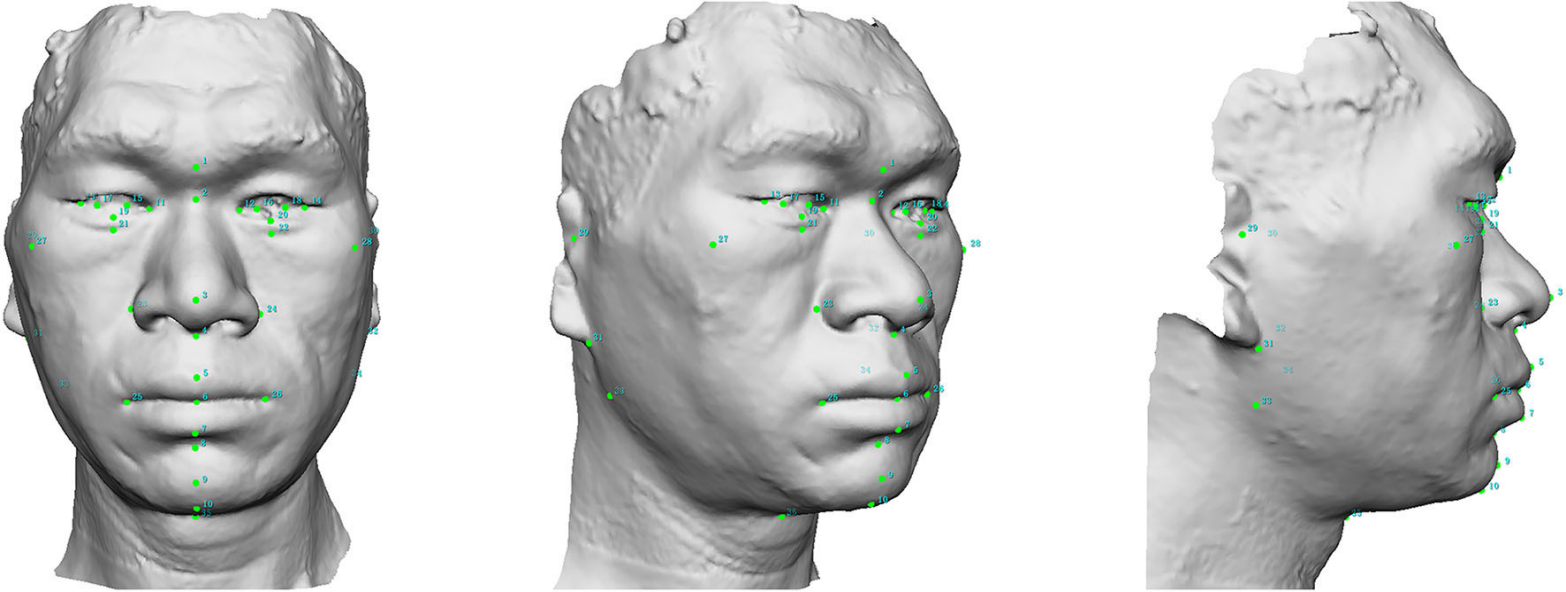
Thirty-five soft tissue landmarks were plotted in each 3D facial image. (1): g, glabella; (2): n, nasion; (3): prn, pronasale; (4): sn, subnasale; (5): ls, labiale superius; (6): sto, stomion; (7): li, labiale inferius; (8): sm, supramentale; (9): pg, pogonion; (10): gn, gnathion; (11, 12): en, endocanthion; (13, 14): ec, ectocanthion; (15, 16): im, iridion mediale; (17, 18): il, iridion laterale; (19, 20): ii, iridion inferius; (21, 22): or, inferior orbital groove; (23, 24): al, alare; (25, 26): ch, chelion; (27, 28): zy, zygion; (29, 30): tr, tragion; (31, 32): sba, subaurale; (33, 34): go, gonion; (35): cm, cervico-mandibular point.

A total of 35 landmarks were adopted in the presented study as defined and illustrated in Figure 1, Supplemental Tables 1, 2. Fifty-five linear, angular, and index parameters were then measured on each face (Supplemental Tables 3–6, Supplemental Figure 1). In addition, semi-perimeters passing three horizontal planes were measured. For this purpose, we adjusted each head to Frankfort Horizontal Plane (FH), marked bilateral preaurale (pra, the most anterior point of the ear at the base of the trgus), and removed the regions outside the two plumb lines passing through bilateral preaurale (Supplemental Figure 2). The three semi-perimeters were defined as pass transglabellar plane (PTGP), pass midfacial plane (PMFP), and pass transverse nasal plane (PTNP). In all, parameters counted up to 58.

### Statistical Analysis

#### Univariate Analysis

To examine the differences between the average values of each individual variable in each group, we first conducted a two-way analysis of variance (ANOVA) with gender (male/female) and disease condition (patient/control) as factors. Homogeneity of variances between subgroups was checked and confirmed by the Levene's test. The ANOVA provided tests on whether the means of each individual variable changed as a function of gender and disease condition (two main effects), and whether non-additive changes existed when both factors varied (one gender^*^disease condition interaction effect). Next, to directly examine the simple main effect of disease condition within each gender, we complemented the above ANOVA by *post-hoc* independent sample *t*-tests between the patient and the control groups. To account for the number of tests conducted for each comparison over multiple variables, we applied Bonferroni correction for the determination of statistical significance. Namely, the threshold for significance is the regular level (alpha = 0.05) divided by the number of tests (58 in our case), which equals 8.62e-4.

#### Multivariate Analysis

##### Linear discriminant analysis (LDA)

LDA is a multivariate statistical technique widely used in machine learning of large-scale, multi-dimensional data. The goal of an LDA is to seek and specify linear combinations (called discriminant functions) of the input dimensions that maximize the differentiation between two groups (i.e., “training”). The discriminant functions could further be used to predict the group membership (patient vs. control) of any new data points that were not included in the training stage. By comparing the predicted vs. the true group membership of the new data points (i.e., “testing”), we could assess the accuracy of the predictions and, in turn, determine if there is any difference at the multi-dimensional level between the two groups. Importantly, the out-of-sample nature of the testing overcomes the pitfall of over-fitting and is a strict and rigorous test of the prediction performance of the discriminant functions.

To fully utilize the entire sample, we performed a leave-one-out cross-validation procedure popular in machine learning. Specifically, we trained the discriminant functions via LDA on n-1 subjects (with n being the total number of subjects with both groups combined), generated the predicted group for the remaining subjects, and compared the prediction with the true group membership. We then iterated this process for n times, looping over all the n subjects in the sample. This process yielded the out-of-sample predicted group membership for the entire sample and the overall prediction accuracy as a proportion between 0 and 1.

The null hypothesis (H_0_) is that there is no information indicative of whether a subject belongs to the patient or the control group in the facial morphological measurements. Under such hypothesis, the prediction accuracy of the above procedure would be at chance level (0.5). To perform statistical inference against this hypothesis, we performed a permutation procedure with 10,000 samples with the grouping labels randomly shuffled. The *p*-value of this prediction accuracy was obtained by comparing against the null distribution generated by the permutation procedure.

After establishing that the LDA is able to utilize the multivariate information in the facial morphological variables to predict disease states, we further interrogated the contributions of individual variables to the differentiation of disease states, in which, we examined the loading coefficients of the individual variables and identified those that contributed the most (i.e., having loading coefficients of the greatest absolute magnitude) to the separation between healthy and disease states. Note that this analysis was based on an LDA with the entire sample, unlike the previous leave-one-out procedure. Importantly, the standardization (z-scoring) of the individual variables was performed prior to the LDA. As a result, all variables were transformed to a common scale so that their LDA loading coefficients were comparable irrespective of potential differences of the raw scales these variables were on originally.

Given the known differences between male and female identified by the univariate analysis, this analysis above was carried out separately in both genders. All the described statistical analyses were performed using the statistical package R (version 3.5.1) and the integrated development environment RStudio (version 1.1.456). The homogeneity of covariance of LDA was checked and verified by Box's *M*-tests.

## Results

### Univariate Analysis

Results of the two-way ANOVA and the *post hoc t*-tests between the disease conditions within each gender are presented in Table 1.

**Table 1:** Results of the two-way ANOVA and the *post hoc t*-tests.

**Variable name (full)**	**Variable name (acronym)**	**Male control (*n* = 34) mean ± SD**	**Male patient (*n* = 34) mean ± SD**	**Female control (*n* = 28) mean ± SD**	**Female patient (*n* = 28) mean ± SD**	**ANOVA disease main effect *p*-value**	**ANOVA gender main effect *p*-value**	ANOVA disease*gender *p*-value	***T*-test male patient vs. control *p*-value**	***T*-test female patient vs. control *p*-value**
Morphological face height	MFH	122.15 ± 7.79	136.57 ± 7.26	113.78 ± 4.30	122.53 ± 6.34	1.93E-11	4.00E-10	7.21E-02	6.48E-08	1.01E-04
Lower facial height	LFH	71.14 ± 6.19	82.39 ± 5.33	65.23 ± 3.46	74.69 ± 5.63	6.95E-13	4.86E-07	4.70E-01	4.79E-08	5.79E-06
Face breadth	FB	127.66 ± 7.80	144.54 ± 7.07	125.38 ± 6.41	130.31 ± 9.77	2.40E-09	1.67E-05	1.34E-03	1.20E-09	1.04E-01
Bigonial breadth	BB	136.90 ± 8.52	140.19 ± 9.36	128.90 ± 6.62	128.87 ± 7.81	3.09E-01	2.97E-06	3.88E-01	2.20E-01	9.90E-01
Bitragal width	BtW	155.03 ± 6.11	165.73 ± 7.91	147.34 ± 4.79	149.51 ± 4.89	2.73E-06	3.60E-12	4.09E-03	7.02E-06	2.16E-01
Binocular width	BnW	93.60 ± 4.10	100.22 ± 4.77	87.88 ± 2.96	91.63 ± 5.44	6.48E-07	8.59E-10	1.63E-01	8.54E-06	2.34E-02
Intercanthal width	ICW	41.56 ± 2.73	42.21 ± 2.96	38.10 ± 1.90	38.54 ± 3.04	3.63E-01	2.51E-07	8.59E-01	4.39E-01	6.33E-01
Ocular width	OW	26.62 ± 1.77	29.38 ± 1.81	25.46 ± 1.29	27.05 ± 1.78	9.15E-08	2.97E-05	1.39E-01	4.51E-06	7.48E-03
Nose height	NH	52.89 ± 3.58	57.17 ± 3.77	50.09 ± 2.22	50.34 ± 4.68	2.29E-03	2.26E-07	1.96E-02	2.79E-04	8.50E-01
Nose length	NL	45.35 ± 3.22	50.97 ± 4.75	41.65 ± 2.57	42.06 ± 4.41	1.68E-04	7.58E-10	4.65E-03	3.26E-05	7.52E-01
Nose width	NW	43.24 ± 2.30	51.21 ± 4.14	40.21 ± 2.50	45.59 ± 2.25	1.01E-15	2.09E-08	6.48E-02	1.92E-09	4.86E-07
Nasal depth	ND	19.14 ± 1.87	21.19 ± 2.40	18.60 ± 1.82	19.56 ± 2.33	1.31E-03	2.99E-02	2.68E-01	2.34E-03	2.02E-01
Mouth width	MW	49.49 ± 3.21	56.39 ± 3.94	47.84 ± 3.34	53.50 ± 4.55	1.11E-10	1.05E-02	4.76E-01	7.11E-08	4.20E-04
Total upper lip height	TULH	24.89 ± 2.79	28.64 ± 3.12	22.47 ± 1.54	26.97 ± 3.36	1.57E-08	2.37E-03	5.65E-01	9.47E-05	8.14E-05
Philtrum length	PL	17.86 ± 2.34	19.40 ± 3.51	15.94 ± 1.53	18.24 ± 3.45	5.47E-03	2.21E-02	5.69E-01	8.72E-02	2.40E-02
Upper vermilion height	UVH	9.50 ± 1.97	11.80 ± 2.30	8.25 ± 0.90	10.95 ± 1.09	3.61E-08	1.22E-02	6.38E-01	7.01E-04	2.18E-08
Lower vermilion height	LVH	8.85 ± 1.81	13.50 ± 2.29	8.28 ± 0.99	11.53 ± 1.91	9.95E-15	4.08E-03	1.06E-01	1.85E-09	4.20E-06
Upper vermilion curve length	UVCL	10.17 ± 2.34	12.95 ± 2.84	8.69 ± 0.96	12.07 ± 1.41	2.63E-08	1.93E-02	5.46E-01	7.66E-04	1.83E-08
Lower vermilion curve length	LVCL	9.48 ± 2.22	15.18 ± 3.16	8.75 ± 1.25	12.76 ± 2.66	2.58E-13	7.44E-03	1.43E-01	1.53E-08	1.92E-05
Mandible curve length	MCL	73.81 ± 7.19	87.38 ± 8.05	72.35 ± 4.08	82.51 ± 8.45	1.62E-10	6.19E-02	3.09E-01	3.17E-07	2.80E-04
Facial length index	FLI	95.87 ± 6.44	94.60 ± 4.83	90.97 ± 5.89	94.53 ± 8.55	6.25E-01	9.69E-02	1.06E-01	4.55E-01	1.82E-01
Mandibulo facial index	MFI	107.37 ± 5.44	97.13 ± 6.88	103.01 ± 6.87	99.24 ±7.05	2.17E-06	4.56E-01	3.44E-02	1.50E-06	1.36E-01
Intercanthal index	II	44.41 ± 2.39	42.12 ± 2.20	43.37 ± 1.77	42.04 ± 1.82	1.68E-04	2.52E-01	3.24E-01	1.54E-03	4.55E-02
Nasal length index	NLI	43.35 ± 2.44	41.88 ± 1.96	44.04 ± 1.73	41.09 ± 3.39	2.97E-04	9.32E-01	1.89E-01	2.90E-02	5.18E-03
Nasal width index	NWI	82.11 ± 6.91	89.76 ± 7.30	80.46 ± 6.66	91.40 ± 10.70	3.21E-06	9.98E-01	3.68E-01	6.90E-04	1.89E-03
Labial index	LI	33.74 ± 7.22	41.51 ± 7.80	32.08 ± 4.97	39.30 ± 5.37	3.81E-06	2.12E-01	8.60E-01	1.07E-03	4.46E-04
Upper lip length index	ULLI	34.97 ± 2.08	34.30 ± 3.22	34.48 ± 1.99	36.06 ± 2.79	6.64E-01	2.93E-01	6.40E-02	4.10E-01	7.56E-02
Lower lip length index	LLLI	27.01 ± 3.38	28.27 ± 2.96	27.13 ± 1.76	28.35 ± 3.70	7.81E-02	8.93E-01	9.75E-01	1.86E-01	2.49E-01
Chin height index	CHI	39.29 ± 3.33	38.32 ± 3.42	38.54 ± 2.68	36.79 ± 4.46	1.09E-01	1.61E-01	6.32E-01	3.36E-01	1.92E-01
Iridio chelial index	ICI	107.57 ± 8.76	104.97 ± 7.85	103.67 ± 7.60	99.64 ± 9.15	9.65E-02	1.88E-02	7.13E-01	2.95E-01	1.86E-01
Endocanthal alar index	EAI	96.27 ± 7.08	82.84 ± 7.73	95.14 ± 8.04	84.70 ± 7.45	5.35E-10	8.35E-01	3.92E-01	2.10E-07	6.41E-04
Alar chelial index	ACI	87.71 ± 7.09	91.10 ± 8.34	84.18 ± 4.17	85.68 ± 6.97	1.04E-01	6.93E-03	5.60E-01	1.46E-01	4.68E-01
Labio orbital triangle index	LOTI	104.95 ± 5.29	107.97 ± 7.33	104.01 ± 3.10	107.65 ± 5.35	1.27E-02	6.30E-01	8.13E-01	1.17E-01	2.72E-02
Naso orbital triangle	NOT	81.80 ± 3.72	82.29 ± 4.39	81.55 ± 2.17	84.48 ± 3.28	7.18E-02	2.47E-01	1.45E-01	6.87E-01	6.15E-03
Naso chelial triangle	NCT	80.89 ± 5.61	80.86 ± 7.80	83.66 ± 4.27	81.78 ± 7.01	5.91E-01	2.16E-01	5.34E-01	9.89E-01	3.70E-01
Supraorbital depth	SoD	129.22 ± 3.88	135.74 ± 6.06	120.20 ± 4.26	122.25 ± 4.77	6.03E-05	1.84E-15	5.00E-02	1.02E-04	2.08E-01
Upper facial depth	UFD	124.76 ± 3.40	130.34 ± 5.38	116.44 ± 4.29	117.69 ± 4.17	2.70E-04	4.47E-16	3.57E-02	1.57E-04	4.09E-01
Orbito tragial depth	OTD	89.25 ± 4.64	93.61 ± 5.09	83.13 ± 4.46	85.44 ± 5.57	2.39E-03	2.09E-08	3.68E-01	3.96E-03	2.07E-01
Labio tragial depth	LTD	120.05 ± 4.83	128.46 ± 7.17	108.38 ± 4.50	114.43 ± 6.17	2.96E-07	1.39E-14	3.83E-01	3.59E-05	3.71E-03
Middle facial depth	MFD	132.90 ± 3.94	142.17 ± 7.05	123.86 ± 4.95	127.73 ± 4.54	1.38E-07	1.57E-14	3.12E-02	3.67E-06	2.86E-02
Sublabial depth	SD	145.27 ± 5.01	156.92 ± 9.01	134.34 ± 5.48	141.91 ± 6.23	7.49E-09	2.90E-12	1.94E-01	4.78E-06	1.01E-03
Lower facial depth	LFD	155.61 ± 6.06	169.90 ± 9.38	145.18 ± 6.87	152.94 ± 7.47	3.12E-09	2.91E-11	6.67E-02	3.89E-07	4.68E-03
Gonion tragial distance	GTD	61.06 ± 6.70	73.69 ± 13.28	58.86 ± 5.59	61.33 ± 6.09	7.71E-05	6.84E-04	1.56E-02	2.79E-04	2.42E-01
Gonion gnathion distance	GGD	112.00 ± 7.24	117.84 ± 7.90	103.88 ± 6.89	110.27 ± 6.60	4.22E-04	1.16E-05	8.69E-01	1.23E-02	1.19E-02
Labiale superius Esthetic plane distance	LSEPD	−0.30 ± 2.12	−1.09 ± 2.23	−1.75 ± 1.69	0.88 ± 2.90	2.33E-01	6.21E-01	1.60E-03	2.30E-01	4.52E-03
Labiale inferius Esthetic plane distance	LIEPD	0.74 ± 2.85	2.26 ± 2.58	−0.88 ± 2.42	3.38 ± 3.76	1.41E-04	7.10E-01	4.49E-02	6.42E-02	7.92E-04
Labiale inferius to E distance minus Labiale superius to E distancee superius to E distance	LIE_LSE	1.04 ± 1.52	3.35 ± 2.25	0.88 ± 1.20	2.50 ± 2.66	2.31E-05	2.68E-01	4.58E-01	2.24E-04	3.71E-02
Glabella TVL distance	GTVLD	−2.00 ± 2.69	−2.52 ± 3.79	−1.48 ± 2.13	−4.81 ± 3.73	2.29E-02	2.30E-01	5.97E-02	5.90E-01	4.90E-03
Pronasale TVL distance	PrTVLD	14.99 ± 1.44	17.67 ± 2.20	14.02 ± 1.67	14.04 ± 2.17	3.90E-04	1.18E-06	3.15E-03	1.88E-05	9.73E-01
Pogonion TVL distance	PoTVLD	−8.45 ± 3.82	−10.96 ± 6.15	−2.32 ± 4.37	5.79 ± 6.57	7.23E-02	6.07E-01	7.51E-01	1.05E-01	3.88E-01
Nasofrontal angle	NFrA	144.00 ± 6.74	132.65 ± 9.10	148.06 ± 5.12	141.75 ± 5.67	1.54E-07	1.28E-04	1.26E-01	2.14E-05	2.49E-03
Nasomental angle	NmA	134.61 ± 5.21	130.17 ± 4.03	134.75 ± 3.91	130.69 ± 5.61	1.50E-04	7.64E-01	8.65E-01	2.44E-03	2.48E-02
Naso facial angle	NFA	28.17 ± 3.64	32.13 ± 2.67	27.94 ± 2.49	32.31 ± 3.66	1.84E-07	9.70E-01	7.75E-01	1.41E-04	5.20E-04
Columella labial angle	CLA	97.91 ± 7.85	90.09 ± 11.91	103.63 ± 6.23	101.44 ± 8.60	9.41E-03	1.21E-04	1.84E-01	1.22E-02	4.17E-01
Facial angle	FA	169.22 ± 4.75	168.39 ± 5.66	168.44 ± 4.63	166.19 ± 6.39	2.50E-01	2.32E-01	5.67E-01	5.95E-01	2.64E-01
Pass transglabellar plane	PTGP	283.45 ± 9.79	310.76 ± 11.12	266.42 ± 17.92	269.33 ± 12.74	7.29E-08	2.88E-15	8.85E-05	2.94E-11	6.01E-01
Pass midfacial plane	PMFP	288.68 ± 10.24	314.04 ± 10.58	270.35 ± 12.83	278.98 ± 11.50	2.51E-10	4.15E-16	1.72E-03	1.73E-10	5.42E-02
Pass transverse nasal plane	PTNP	293.61 ± 15.12	316.81 ± 15.14	286.32 ± 16.34	292.29 ± 15.13	1.52E-05	2.55E-05	1.74E-02	4.96E-06	2.92E-01

*Highlights means variables show statistically significant differences*.

A large number of facial morphological variables show a significant main effect of disease condition (38 out of the 58 we examined in this study). The directions of the difference are mostly those patients had higher average values in these morphological variables (32 out of 38 variables), except for 6 variables (4 index and 2 angular variable) that showed an opposite difference direction, which are mandibulo-facial index, intercanthal index, nasal length index, endocanthal alar index, nasofrontal angle, and nasomental angle, respectively.

Similarly, many facial morphological variables show a significant main effect of gender (26 out of 58), with 21 of them overlapping with those with a significant main effect of disease conditions. The majority of these variables have higher values in males than in females, with notable exceptions in two angles (nasofrontal angle and columella labial angle). Almost all morphological variables show no significant interaction between disease condition and gender.

Consistent with the above observations, *post-hoc* tests between disease conditions in each gender reveal a larger number of morphological variables (33 of 58 in males, 14 of 58 in females, with 12 overlapping in both genders) that show statistically significant differences (also see Table 1).

**Figure 2:**
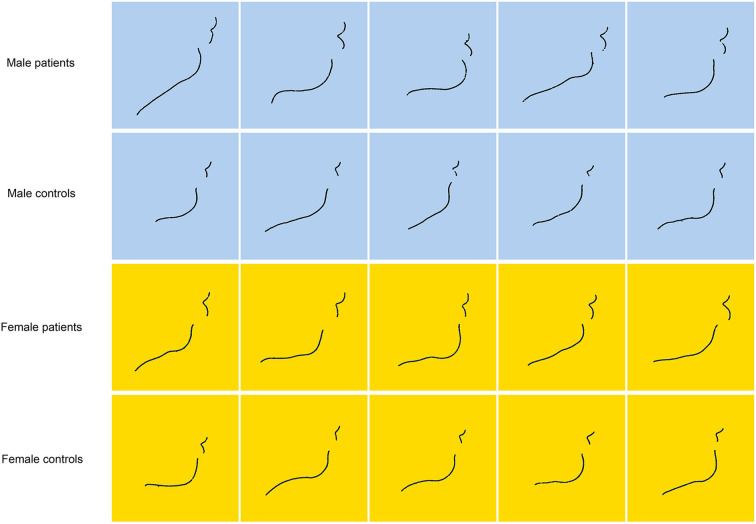
The profile curves of vermilion and mandible of patients and their matched controls. Each row represents a male patient, a female patient and their paired healthy controls. The blue background represents males and the yellow background represents females. It shows that vermilion of acromegalic patients are significantly thickened and everted, which is more obvious in the lower lip.

Comprehensively considering the above ANOVA and *t*-test results, the facial changes of acromegalic patients can be summarized in Table 2, which are presented based on the common changes of patients, the unchanged parts, and differences of male and female patients. In order to visualize the shape of lips and chin intuitively, the profile curve of each subject's vermilion and mandible was automatically depicted and measured. Figure 2 shows the profile curves of patients and their matched controls.

**Table 2:** Summary of facial morphological changes of acromegalic patients and differences between genders based on the univariate analysis results.

	**Upper face**	**Midface**	**Lower face**	**Angles of profile**	**The whole face**
Common changes of acromegalic patients	Binocular width ↑ Ocular width ↑ Intercanthal index ↓	Nose width ↑ Nasal width index ↑ Nasal width index ↓	Mouth width ↑ Total upper lip height ↑ Upper vermilion height ↑ Lower vermilion height ↑ Upper vermilion curve length ↑ Lower vermilion curve length ↑ Mandible curve length ↑ Gonion-tragial distance ↑ Gonion-gnathion distance ↑ Labial index ↑ Labialeinferius-E distance ↑	Nasofrontal angle ↓ Nasomental angle ↓ Naso facial angle ↑ Columella labial angle ↓	Morphological face height ↑ Lower facial height ↑ Face breadth ↑ Bitragal width ↑ Mandibulo facial index ↓ Nasal length index ↓ Endocanthal alar index ↓
No significant difference between patients and healthy controls	Intercanthal width -Glabella-TVL distance -	Nasal depth -	Bigonial breadth - Philtrum length - Pogonion-TVL distance - Labialesuperius-E distance -	Facial angle -	Facial length index - Chin height index - Iridiochelial index - Alar chelial index -
Differences between male and female patients					The facial depths and the head circumferences of male patients increase at all levels, while those of the female patients do not

*↑, increase; ↓, decrease; -, no significant difference; TVL, subnasale true vertical line; E, Esthetic plane*.

### Multivariate Analysis

#### Linear Discriminant Analysis

To directly test the possibility of using combinations of the facial morphological measures to predict disease conditions, we performed linear discriminant analysis (LDA) on each gender separately. Using a leave-one-out cross-validation procedure, we obtained overall out-of-sample prediction accuracy of the LDA algorithm, and the results are presented in Table 3. In both genders, LDA achieves very high prediction accuracies (92.86% in females and 75% in males). We further applied a permutation procedure to establish the statistical significance of such prediction accuracies against the chance level, and both turned out to be highly significant (*p* < 0.0001 for females and *p* < 0.001 for males).

**Table 3:** Overall out-of-sample prediction accuracy of the LDA algorithm.

**Prediction/fact**	**Control**	**Patient**
**Female: LDA out-of-sample accuracy**		
Control	28	4
Patient	0	24
Overall prediction accuracy		0.9286
*p*-value against H_0_		<0.0001
prediction/fact	Control	Patient
**Male: LDA out-of-sample accuracy**		
Control	26	9
Patient	8	25
Overall prediction accuracy	0.75	
*p*-value against H_0_	<0.001	

**Figure 3:**
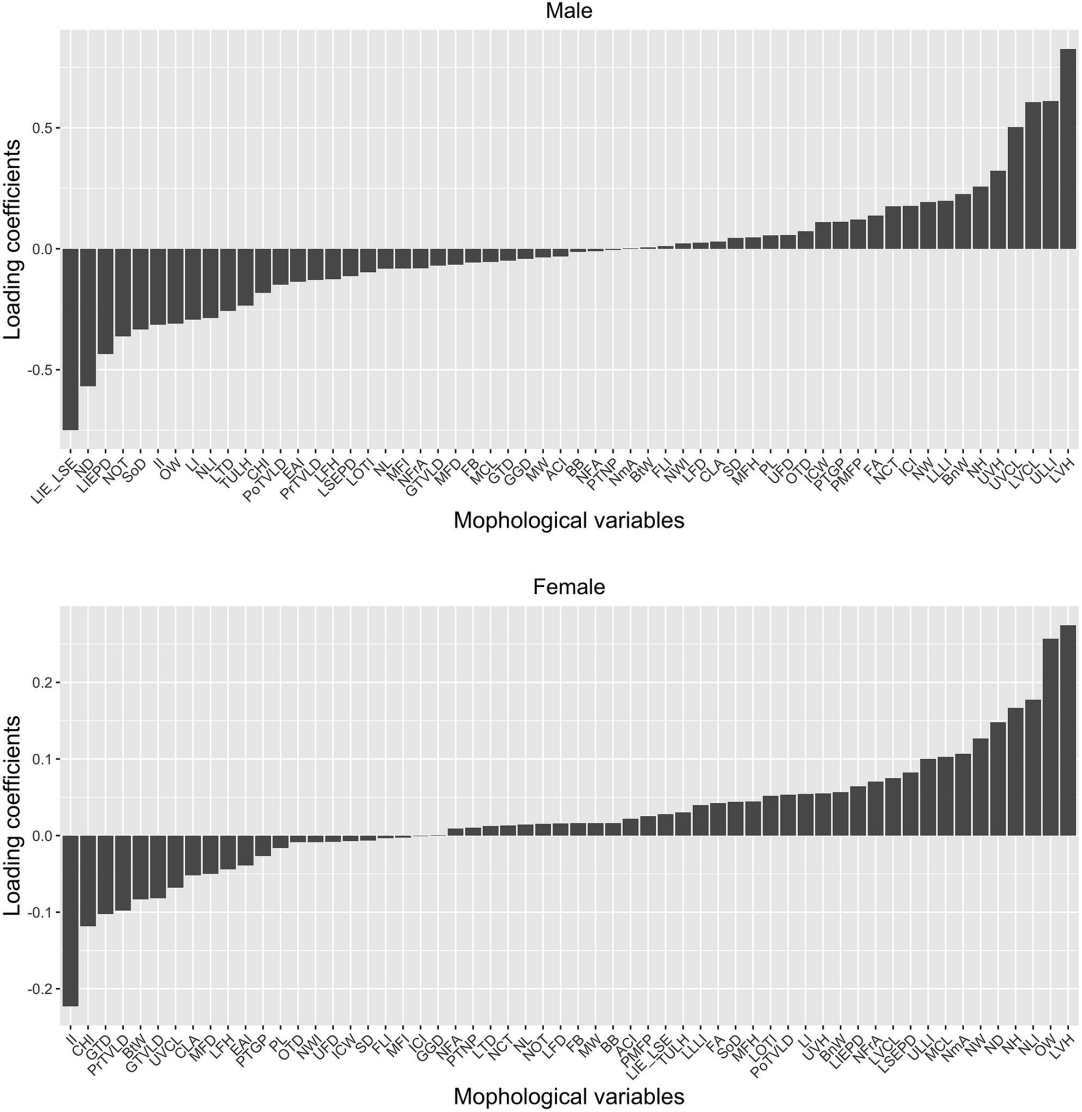
The LDA loading coefficients. Variables with loading coefficients of larger absolute value (regardless of the sign) have higher weights and thus greater contributions to the differentiation between the healthy and the disease states. As shown in this Figure, only a few facial morphological variables play a vital role in disease prediction. Also, the facial morphological variables have distinct profiles in males vs. females in terms of their contribution to the classification. LIE_LSE, Labiale inferius to E distance minus Labiale superius to E distance; ND, Nasal depth; LIEPD, Labiale inferius-Esthetic plane distance; NOT, Naso-orbital triangle; SoD, Supraorbital depth; II, Intercanthal index; OW, Ocular width; LI, Labial index; NLI, Nasal length index; LTD, Labio-tragial depth; TULH, Total upper lip height; CHI, Chin height index; PoTVLD, Pogonion-TVL distance; EAI, Endocanthal-alar index; PrTVLD, Pronasale-TVL distance; LFH, Lower facial height; LSEPD, Labiale superius-Esthetic plane distance; LOTI, Labio-orbital triangle index; NL, Nose length; MFI, Mandibulo-facial index; NFrA, Nasofrontal angle; GTVLD, Glabella-TVL distance; MFD, Middle facial depth; FB, Face breadth; MCL, Mandible curve length; GTD, Gonion-tragial distance; GGD, Gonion-gnathion distance; MW, Mouth width; ACI, Alar-chelial index; BB, Bigonial breadth; NFA, Naso-facial angle; PTNP, Pass transverse nasal plane; NmA, Nasomental angle; BtW, Bitragal width; FLI, Facial length index; NWI, Nasal width index; LFD, Lower facial depth; CLA, Columella-labial angle; SD, Sublabial depth; MFH, Morphological face height; PL, Philtrum length; UFD, Upper facial depth; OTD, Orbito-tragial depth; ICW, Intercanthal width; PTGP, Pass transglabellar plane; PMPF, Pass midfacial plane; FA, Facial angle; NCT, Naso-chelial triangle; ICI, Iridio-chelial index; NW, Nose width; LLLI, Lower lip length index; BnW, Binocular width; NH, Nose height; UVH, Upper vermilion height; UVCL, Upper vermilion curve length; LVCL, lower vermilion curve length; ULLI, Upper lip length index; LVH, Lower vermilion height.

In order to identify the relative contributions of the individual morphological variables to such accurate classification, the LDA loading coefficients are presented in Figure 3. As shown in Figure 3, not all facial morphological variables contributed equally to the accurate classification of the subjects into the patient and the control groups. Rather, such classification was primarily driven by a minority of variables. Also, the facial morphological variables have distinct profiles in males vs. females in terms of their contribution to the classification. For instance, in males, the 10 variables with the most contribution are lower vermilion height, upper lip length index, lower vermilion curve length, upper vermilion curve length, upper vermilion height, labiale inferius to E plane distance minus labiale superius to E plane distance, nasal depth, labiale inferius to E plane distance, naso orbital triangle, and supraorbital depth. In females, however, the list includes lower vermilion height, ocular width, nasal length index, nose height, nasal depth, nose width, nasomental angle, intercanthal index, chin height index, and gonion tragial distance, which has minor overlap with that of males. It highlights the existence of the gender difference in the diagnosticity of the morphological variables for acromegaly.

## Discussion

Acromegaly, a rare chronic disease, is characterized by occult onset, slow progression that eventually leads to multi-system involvement and shortened life expectancy (3, 4). The disease has always been under-recognized or delayed in diagnosis for many years, and its incidence has also been underestimated (17–20). The long course of the disease will accumulate irreversible multi-system damage to the patients. Therefore, early diagnosis is essential for implementing early interventions and improving the prognosis of patients.

Acral enlargement and facial changes are the most common manifestations of acromegaly, which also are the important basis for the diagnosis of the disease (1, 10, 11). For many years, however, such manifestations have little assistance in clinical practices. In most cases, patients don't visit doctors until there have been obvious facial changes. Endocrinology specialists often identify, with unaided eyes, their facial features before drawing preliminary diagnostic conclusions. Why are facial manifestations hardly utilized to its fullest? Information from traditional facial morphological detection is limited and it's impossible to build a data system for clinicians to refer to.

Previous studies on the measurement of maxillofacial parameters in acromegalic patients were mostly focused on their airway obstruction and occlusion problems, and adopted the traditional lateral X-ray cephalometry (21–25). The findings of studies were largely identical but with minor differences. Kunzler and Farmand (21) concluded that statistically significant changes between acromegalic patients and healthy controls could be found only in the mandible. The acromegalic patients had mandibular protrusion and increased mandibular length. The ascending ramus, as well as the body of the mandible, were both elongated. There were no differences in the position of the maxilla between the patients and those in the healthy control group according to their study. The results of Hochban et al. (22) are in line with previous results, revealing that major skeletal changes in acromegalic patients could be found only in the mandible. Dostalova et al. (23) stated that the greatest anomaly was seen in the mandible, contrary to previous studies, which observed the retroposition of the maxilla in acromegalic patients. Based on previous studies, it is generally believed that the maxillofacial changes in acromegalic patients include mandibular protrusion, mandibular lengthening, malocclusion, and mandibular angle enlargement etc. As can be seen, lateral X-ray cephalometry is only available to measure skeletal changes in profile. A large amount of information of the front look and soft tissues has been lost. However, such information is exactly what's necessary for the disease's diagnosis. Nowadays, the application of 3D imaging techniques in clinical practices is on the rise. 3D endoscopy, 3D-printing-model-aided operation, 3D-image-aided operation design are all developed from 3D imaging techniques. The advantage of 3D imaging lies in its revivification of the real spaciousness of objects. This frees observers from the restriction of a single visual angle and allows them to get the whole picture of objects from different angles. In this study, 3D imaging techniques were first used to make a comprehensive analysis of facial features of acromegalic patients; then combinations of facial variables were obtained by using a machining learning technique to verify the feasibility of using these combinations for patient identification.

Using a wealth of data generated by 3D imaging techniques, we present robust quantitative evidence showing that acromegaly patients have specific sets of facial morphological features that are distinct from healthy controls. According to the results of univariate analysis presented in Tables 1, 2, some important inferences can be obtained, as shown in Table 4. Consistent with the previous studies, the measurements obtained in this study also showed that the mandibular length increases, and that both the body and the ascending ramus of the mandible were lengthened. Moreover, we believed that the lengths of the patients' mandible and face increases in proportion. Contrary to previous studies, it was found through the comprehensive analysis of linear and angular parameters that the patients don't present mandibular protrusion alone; that is to say, it may also be simultaneously accompanied by the forward shift in the anterior nasal spine (maxilla). In addition, a series of inferences were also obtained, covering the eyes, nose, mouth, and the whole face, as well as their relationships. The widening of nasal width and thickening of the lips are very significant in acromegalic patients. As showed in Figure 2, vermilion of acromegalic patients are significantly thickened and everted, which is more obvious in the lower lip.

**Table 4:** Inferences of common characteristics in acromegalic patients according to the results of univariate analysis.

	**Inferences**	**Reasons**
Upper face	• The width on the eye level also found to be widened in the patients, which are manifested by increased ocular width and binocular width, but there is no significant change in intercanthal width, that is, the widening on the eye level are not caused by the widening of the nasal bone.	Binocular width ↑, Ocular width ↑, Intercanthal width-, Intercanthal index ↓
Midface	• Nasal changes in the patients are mainly characterized by widened nasal width, but the changes in nasal length are not as significant as that in nasal width. • The nasal dorsum heightens in the patients	Nose width ↑, Nasal width index ↑, Nasal width index ↓ Naso facial angle ↑
Lower face	• Both the body and ascending ramus of the mandible were lengthened • The patients' lips are significantly thickened and everted, the change mainly occurs in the vermilion, and is more obvious in the lower lip	Gonion-tragial distance ↑, Gonion-gnathion distance ↑ Total lip height ↑, Vermilion height ↑, Vermilion curve length ↑, Labial index ↑, Labialeinferius-E distance ↑, Philtrum length -, Labialesuperius-E distance -
The whole face and proportions	• The facial length and width of the patients increase in proportion. • The mandible and facial length of the patients increase in proportion. • The nasal and oral widths of the patients widen in proportion. • The facial widths of the patients widen above the zygion level, but there is no significant change in bigonial breadth. • The patients don't present frontal or mandibular protrusion alone; it may also be simultaneously coupled with the forward shift in the anterior nasal spine (maxilla).	Morphological face height ↑, Face breadth ↑, Facial length index- Morphological face height ↑, Lower facial height ↑, Chin height index- Nose width ↑, Mouth width ↑, Alar chelial index- Face breadth ↑, Bitragal width ↑, Bigonial breadth-, Mandibulo facial index ↓ Nasomental angle ↓, Facial angle -, Glabella-TVL distance -, Pogonion-TVL distance -

*↑, increase; ↓, decrease; -, no significant difference; TVL, subnasale true vertical line; E, Esthetic plane*.

In addition to the common changes, many measured variables of male patients were significantly larger than those of female patients, such as facial breadth, depth, and semi-perimeter at all levels. This was consistent with clinical observation, which revealed that many male patients showed more obvious abnormalities than female patients in height, body shape, or facial appearance. Given the lack of statistically significant interaction effects between disease and gender for many variables, disease condition does not seem to exaggerate or dampen such gender differences. These differences might be mainly caused by the original differences in the facial appearance between both genders, that is, many facial variables of normal males are generally higher than those of females, which is consistent with previous research results (26–28).

Using linear discriminant analysis, a multivariate classification technique from machine learning, we show that the combination of these facial morphological features can be used to predict disease condition to a high accuracy in both genders. These morphological features are not equally informative in predicting disease status; rather, a subset of these features contributed to most of the predictive power (by having the largest loadings on the linear discriminant function), and gender differences in the relative diagnostic importance of morphological features also exist. This provides useful hints for designing more gender-appropriate diagnostic criteria for acromegaly. The differential loadings across the morphological variables we examined also reveal intriguing structure in the relationship between these variables. Our findings calls for future studies using similar approaches to address limitations of the traditional way of examining a small number of morphological features one at a time.

By presenting carefully-designed analyses of a large number of facial morphological variables in both acromegaly patients and matched controls, we provide a comprehensive overview of what features are different and what are not, as well as differences between genders. Such quantitative insights are essential to understand the underlying physiological processes that drive such measurable changes. With the increasing number of research conducted in a larger population in the future, there will be more data available. This will contribute to the development of facial feature data system for the disease. Such data will also be applicable to the identification of acromegaly and other diseases with facial morphological changes.

A highlight of the research findings is the superb accuracy of out-of-sample prediction of disease conditions using the discriminant analysis. This indicates a promising prospect with respect to the combined application of 3D imaging and machine learning techniques in early detection of acromegaly. Highly effective, comprehensive and accurate, 3D imaging is a good choice for the development of screening tools. Machine learning does a better job than human brain in processing data and some feature aggregations. Compared with personal experience, the combined techniques will be more objective and sensitive. Nowadays, 3D imaging is seeing an increasingly wide application to various fields. For example, some mobile phone apps have been developed to capture 3D facial images. In the future, rapid and automatic 3D imaging may realize the recognition of suspected patients in physical examination institutions or patients' homes. With the improvement in detection accuracy, it will become more likely to identify patients with indistinctive facial changes, which will be helpful to the early detection of the disease. An extra step had been taken in the study to gain a deeper insight into different disease prediction weights of various facial variables. The authors believe that such important variables will play a significant role in future software development.

## Limitations

One limitation of this study is the relatively small sample size. Inaccessibility of self-control is another limitation. That is, the researchers cannot collect 3D images of the patients before the onset, but can only match control subjects according to age, gender, height, body weight, etc. This control selection can be used to investigate the differences between acromegalic patients and the normal population. However, if self-control can be obtained, it will help to more accurately elaborate the prognosis of patients' facial changes under the influence of the disease. In the future, 3D imaging techniques will be used in the self-control study before and after treatment to explore the recovery process of patients' facial morphology, which will be an important supplement to existing literature.

## Data Availability Statement

The datasets generated for this study are available on request to the corresponding author.

## Ethics Statement

The studies involving human participants were reviewed and approved by the Institutional Review Board at Peking Union Medical College Hospital, Chinese Academy of Medical Sciences. The patients/participants provided their written informed consent to participate in this study. Written informed consent was obtained from the individual(s) for the publication of any potentially identifiable images or data included in this article.

## Author Contributions

TM, XG, BX, and XL designed the study. TM, XG, JH, WL, KD, LG, and ZW recruited the patients and recorded clinical information. TM conducted the photography and analyzed the data and drafted the manuscript. XG, XW, XL, and BX revised the manuscript. All authors contributed to the article and approved the submitted version.

## Conflict of Interest

The authors declare that the research was conducted in the absence of any commercial or financial relationships that could be construed as a potential conflict of interest.
