# Supplementary material for: Identifying Facial Features and Predicting Patients of Acromegaly Using Three-Dimensional Imaging Techniques and Machine Learning
Source: Front Endocrinol (Lausanne). 2020 Jul 29;11:492. doi: 10.3389/fendo.2020.00492 (PMC7403213; doi:10.3389/fendo.2020.00492)
Supplement: Supplementary file 1 [file Data_Sheet_1.PDF]

Supplemental Table 1 Facial landmarks

Land mark	Definition	Number
glabella (g)	the smooth elevation of the frontal bone between the eyebrows on the MSP	1
nasion (n)	the apex of the grontonasal angle	2
pronasale (prn)	the tip of the nose	3
subnasale (sn)	the lowest point of the nose on the MSP	4
labiale superius (ls)	the intersection of the MSP with the vermilion border of the upper lip	5
stomion (sto)	the intersection of the MSP with the labial fissure	6
labiale inferius (li)	the intersection of the MSP with the vermilion border of the lower lip	7
supramentale (sm)	the intersection of the MSP with the mentolabial sulcus	8
pogonion (pg)	the most anterior point on the chin	9
gnathion (gn)	the lowest point of the chin on the MSP	10
endocanthion (en)	the medial corner of the eye	11,12
ectocanthion (ec)	the lateral corner of the eye	13,14
iridion mediale (im)	the most medial point on the rim of the iris	15,16
iridion laterale (il)	the most lateral point of the rim of the iris	17,18
iridion inferius (ii)	the most inferior point on the rim of the iris	19,20
inferior orbital groove (or)	the most inferior point on the orbital groove	21,22
alare (al)	the most lateral point of the nasal alar	23,24
chelion (ch)	the corner of the mouth	25,26
zygion (zy)	the widest point of the cheek seen in the frontal view	27,28
cervico-mandibular point (cm)	the intersection of the MSP with the cervico-mandibular angle	35
tragion (tr)	the apex of the tragus	29,30
subaurale (sba)	the inferior pole of the lobe	31,32
gonion (go)	the most inferior, posterior, and lateral point on the external angle of the mandible	33,34

MSP, midsagittal plane.
