# Supplementary material for: Identifying Facial Features and Predicting Patients of Acromegaly Using Three-Dimensional Imaging Techniques and Machine Learning
Source: Front Endocrinol (Lausanne). 2020 Jul 29;11:492. doi: 10.3389/fendo.2020.00492 (PMC7403213; doi:10.3389/fendo.2020.00492)
Supplement: Supplementary file 2 [file Data_Sheet_2.PDF]

Supplemental Table 2 Reference planes

	Plane	Definition
**represented on the lateral view of facial soft tissue**	Frankfort Horizontal Plane (FH)	The plane represents the head in natural anatomical position. One the lateral view of facial soft tissue, this plane can be represented by the line connecting the tragion with the inferior orbital groove.
	Esthetic plane (E)	A line connecting pronasale with pogonion. The plane was often used in assessing lip and nose relations, and also evaluating profile esthetics.
	subnasale true vertical line (TVL)	the plumb line passing through the subnasal in the natural head position
**represented on the frontal view of facial soft tissue**	midsagittal plane (MSP)	divides the face into bilaterally symmetrical right and left sides connecting all midline points from vertex to gnathion
	midfacial plane (MFP)	divides the head into upper and lower halves by a horizontal line tangent to the inferior poles of the irises
	transverse nasal plane (TNP)	a horizontal plane parallel to the MFP and passing through the subnasale
	transglabellar plane (TGP)	a horizontal plane passing through the glabella and marking the upper side of the facial square
