# Supplementary material for: Identifying Facial Features and Predicting Patients of Acromegaly Using Three-Dimensional Imaging Techniques and Machine Learning
Source: Front Endocrinol (Lausanne). 2020 Jul 29;11:492. doi: 10.3389/fendo.2020.00492 (PMC7403213; doi:10.3389/fendo.2020.00492)
Supplement: Supplementary file 3 [file Data_Sheet_3.PDF]

Supplemental Table 3 Linear measurement on the frontal view

	Distance	Abbreviation
**Facial**	Morphological face height (n-gn)	MFH
	Lower facial height (sn-gn)	LFH
	Face breadth (zy-zy)	FB
	Bigonial breadth (go-go)	BB
	Bitragal width (tr-tr)	BtW
**Orbital**	Binocular width (ec-ec)	BnW
	Intercanthal width (en-en)	ICW
	Ocular width (en-ec)	OW
**Nasal**	Nose height (n-sn)	NH
	Nose length (n-prn)	NL
	Nose width (al-al)	NW
	Nasal depth (sn-prn)	ND
**Labial**	Mouth width (ch-ch)	MW
	Total upper lip height (sn-sto)	TULH
	Philtrum length (sn-ls)	PL
	Upper vermilion height (ls-sto)	UVH
	Lower vermilion height (sto-li)	LVH
	Upper vermilion curve length	UVCL
	Lower vermilion curve length	LVCL
**mandible**	Mandible curve length	MCL
