# Supplementary material for: Identifying Facial Features and Predicting Patients of Acromegaly Using Three-Dimensional Imaging Techniques and Machine Learning
Source: Front Endocrinol (Lausanne). 2020 Jul 29;11:492. doi: 10.3389/fendo.2020.00492 (PMC7403213; doi:10.3389/fendo.2020.00492)
Supplement: Supplementary file 4 [file Data_Sheet_4.PDF]

Supplemental Table 4 Angular and index surveying on the frontal view

	Variable	Algorithm	Abbreviation
**Index**	Facial length index	morphological face height/face breadth (n-gn/zy-zy ×100)	FLI
	Mandibulo-facial index	bigonial breadth/face breadth (go-go/zy-zy ×100)	MFI
	Intercanthal index	intercanthal width/binocular width (en-en/ec-ec ×100)	II
	Nasal length index	nose height/morphological face height (n-sn/n-gn ×100)	NLI
	Nasal width index	nose width/nose height (al-al/n-sn ×100)	NWI
	Labial index	vermilion height/mouth width (ls-li/ch-ch ×100)	LI
	Upper lip length index	total upper lip height/lower facial height (sn-sto/sn-gn ×100)	ULLI
	Lower lip length index	total lower lip height/lower facial height (sto-sm/sn-gn ×100)	LLLI
	Chin height index	chin height/lower facial height (sm-gn/sn-gn ×100)	CHI
	Iridio-chelial index	width between iridion mediales/mouth width (im-im/ch-ch ×100)	ICI
	Endocanthal-alar index	width between endocanthions/nose width (en-en/al-al ×100)	EAI
	Alar-chelial index	nose width/mouth width (al-al/ch-ch ×100)	ACI
	Labio-orbital triangle index	ec-li/ec-ec ×100	LOTI
**Angle**	Naso-orbital triangle	ec-sn-ec	NOT
	Naso-chelial triangle	ch-sn-ch	NCT
