# Supplementary material for: Identifying Facial Features and Predicting Patients of Acromegaly Using Three-Dimensional Imaging Techniques and Machine Learning
Source: Front Endocrinol (Lausanne). 2020 Jul 29;11:492. doi: 10.3389/fendo.2020.00492 (PMC7403213; doi:10.3389/fendo.2020.00492)
Supplement: Supplementary file 5 [file Data_Sheet_5.PDF]

Supplemental Table 5 Linear measurement on the lateral view

	Distance	Abbreviation
**Facial depth**	Supraorbital depth (tr-g)	SoD
	Upper facial depth (tr-n)	UFD
	Orbito-tragial depth (tr-ex)	OTD
	Labio-tragial depth (tr-ch)	LTD
	Middle facial depth (tr-sn)	MFD
	Sublabial depth (tr-sm)	SD
	Lower facial depth (tr-gn)	LFD
**Position of mandibular angle**	Gonion-tragial distance (go-tr)	GTD
	Gonion-gnathion distance (go-gn)	GGD
**Measurement based on E plane**	Labiale superius-Esthetic plane distance (ls-E)	LSEPD
	Labiale inferius-Esthetic plane distance (li-E)	LIEPD
	Labiale inferius to E distance minus Labiale superius to E distance [(li-E)-(ls-E)]	LIE_LSE
**Measurement based on TVL**	Glabella-TVL distance (g-TVL)	GTVLD
	Pronasale-TVL distance (prn-TVL)	PrTVLD
	Pogonion-TVL distance (pog-TVL)	PoTVLD

E plane, esthetic plane; TVL, subnasale true vertical line.
