# Supplementary material for: Identifying Facial Features and Predicting Patients of Acromegaly Using Three-Dimensional Imaging Techniques and Machine Learning
Source: Front Endocrinol (Lausanne). 2020 Jul 29;11:492. doi: 10.3389/fendo.2020.00492 (PMC7403213; doi:10.3389/fendo.2020.00492)
Supplement: Supplementary file 6 [file Data_Sheet_6.PDF]

Supplemental Table 6 Angular surveying on the lateral view

Angle	Abbreviation
Nasofrontal angle (g-n-prn)	NFrA
Nasomental angle (n-prn-pg)	NmA
Naso-facial angle (pog-n-prn)	NFA
Columella-labial angle (cp-sn-ls)	CLA
Facial angle (n-sn-pg)	FA
