# Supplementary material for: Identifying Facial Features and Predicting Patients of Acromegaly Using Three-Dimensional Imaging Techniques and Machine Learning
Source: Front Endocrinol (Lausanne). 2020 Jul 29;11:492. doi: 10.3389/fendo.2020.00492 (PMC7403213; doi:10.3389/fendo.2020.00492)
Supplement: Supplementary file 7 [file Data_Sheet_7.PDF]

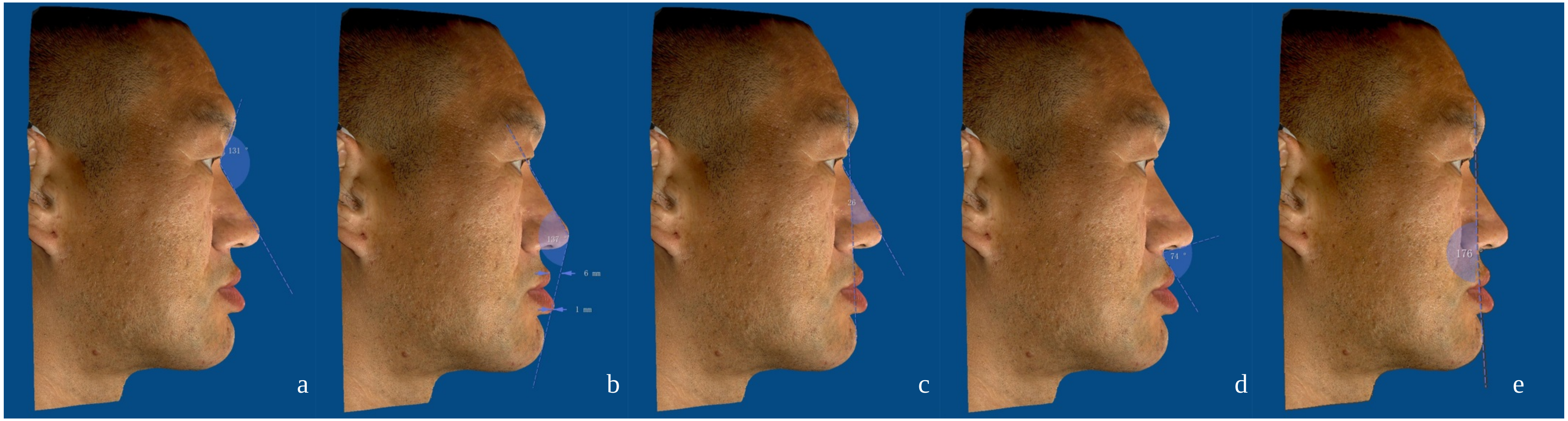

Supplemental Figure 1 Angular surveying on the lateral view

Five angles of facial profile were automatically measured by VECTRA software.

a: Nasofrontal angle (g-n-prn, NFrA); b: Nasomental angle (n-prn-pg, NmA); c: Naso-facial angle (pog-n-prn, NFaA); d: Columella-labial angle (cp-sn-ls, CLA); e: Facial angle (n-sn-pg, FA).
