# Supplementary material for: Identifying Facial Features and Predicting Patients of Acromegaly Using Three-Dimensional Imaging Techniques and Machine Learning
Source: Front Endocrinol (Lausanne). 2020 Jul 29;11:492. doi: 10.3389/fendo.2020.00492 (PMC7403213; doi:10.3389/fendo.2020.00492)
Supplement: Supplementary file 8 [file Data_Sheet_8.PDF]

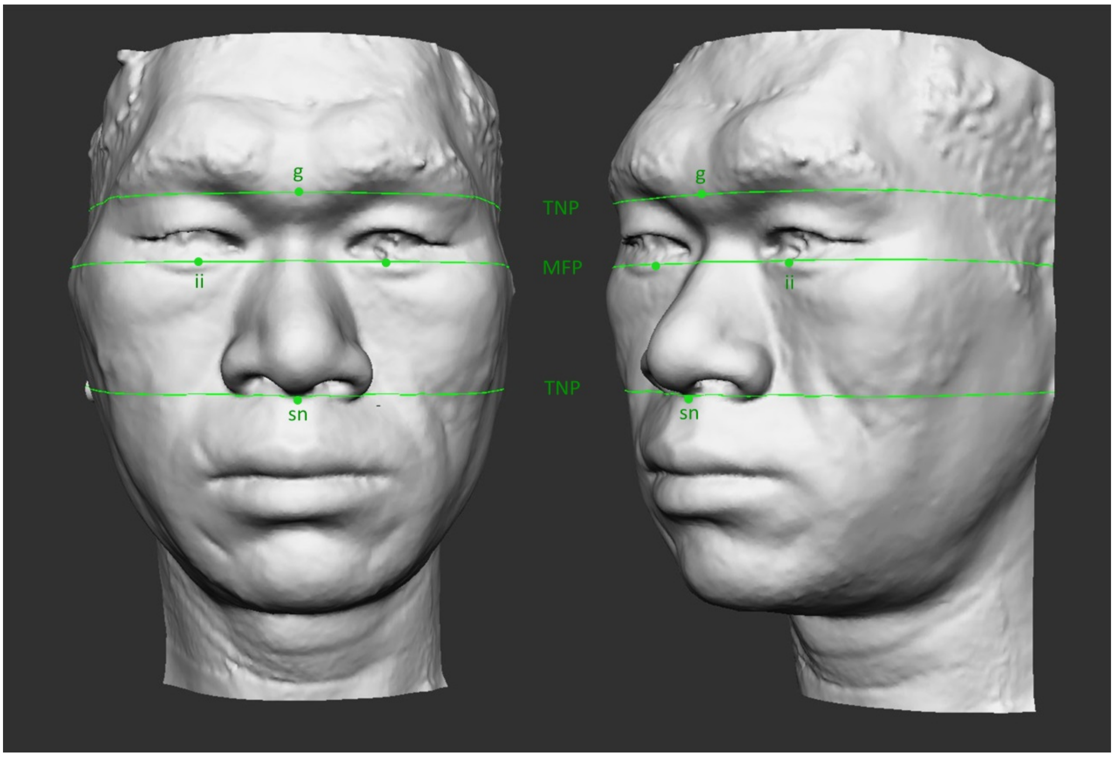

Supplemental Figure 2 Semi-perimeters passing three horizontal planes

TGP, transglabellar plane; MFP, midfacial plane; TNP, transverse nasal plane; g, glabella; ii, iridion inferius; sn, subnasale.
